# Diabetes modifies the cross-sectional association between Healthy Eating Index-2015 and clinically relevant depressive symptoms: a NHANES analysis with an independent hospital-based replication cohort

**DOI:** 10.3389/fnut.2026.1844683

**Published:** 2026-06-17

**Authors:** Hao Sun, Zhenxu Han, Yuantao Qi, Yifan Li

**Affiliations:** 1 Qingdao Hospital, University of Health and Rehabilitation Sciences (Qingdao Municipal Hospital), Qingdao, China; 2 Qingdao Zhanshan Sanatorium, Qingdao, China; 3 Shandong Cancer Hospital and Institute, Shandong First Medical University and Shandong Academy of Medical Sciences, Jinan, China

**Keywords:** clinically relevant depressive symptoms, diabetes, diet quality, dietary patterns, HEI-2015, mental health

## Abstract

**Background:**

Although dietary patterns have been increasingly linked to mental health, evidence regarding the cross-sectional association between overall diet quality and clinically relevant depressive symptoms remains inconsistent. We aimed to examine the association between the Healthy Eating Index-2015 (HEI-2015) and PHQ-9-defined clinically relevant depressive symptoms in a nationally representative U.S. population and to assess whether the findings were reproducible in an independent hospital-based replication cohort, with particular attention to the modifying role of diabetes.

**Methods:**

We analyzed cross-sectional data from 12,462 participants in the National Health and Nutrition Examination Survey (NHANES) 2007–March 2020. A replication analysis was performed in an independent cohort of 1,019 individuals who underwent routine health examinations at the Health Examination Center of the Second Affiliated Hospital of Shandong First Medical University between 2024 and 2025. Clinically relevant depressive symptoms were defined as a Patient Health Questionnaire-9 (PHQ-9) score ≥ 10, and dietary quality was assessed using HEI-2015. The hospital-based HEI-2015 score was derived from questionnaire-based dietary information and should be interpreted as a harmonized approximation rather than an identical replication of the NHANES 24-hour recall-based HEI-2015. Multivariable logistic and linear regression models were used to examine the associations of HEI-2015 with PHQ-9-defined clinically relevant depressive symptoms and with PHQ-9 score. Subgroup analyses and restricted cubic spline models were performed to assess effect modification and dose-response patterns.

**Results:**

In the NHANES cohort, higher HEI-2015 was associated with lower odds of PHQ-9-defined clinically relevant depressive symptoms in the fully adjusted model (OR per 1-point increase = 0.992, 95% CI: 0.984–1.000, *P* = 0.048; approximately OR per 10-point increase = 0.923) and with lower PHQ-9 scores (β per 1-point increase = −0.008, 95% CI: −0.014 to −0.002, *P* = 0.006). These findings were reproduced in the hospital-based replication cohort, in which higher HEI-2015 was also associated with lower odds of clinically relevant depressive symptoms (OR = 0.960, 95% CI: 0.925–0.997, *P* = 0.036; approximately OR per 10-point increase = 0.665) and lower PHQ-9 scores (β = −0.049, 95% CI: −0.077 to −0.022, *P* < 0.001). Diabetes significantly modified these associations in both cohorts. Among individuals without diabetes, higher HEI-2015 was associated with more favorable depression-related outcomes, whereas among those with diabetes, the association was directionally opposite. Restricted cubic spline analyses supported a significant overall association between HEI-2015 and clinically relevant depressive symptoms in both cohorts, without strong evidence of nonlinearity.

**Conclusion:**

Higher dietary quality was cross-sectionally associated with lower odds of PHQ-9-defined clinically relevant depressive symptoms and lower depressive symptom burden, and these associations were reproduced in an independent hospital-based replication cohort. However, the association was consistently modified by diabetes, with an opposite-direction association observed among diabetic individuals. These findings should be interpreted cautiously because of the cross-sectional design, possible reverse causation, dietary measurement differences between cohorts, and residual confounding. Prospective and interventional studies are needed to clarify whether and how metabolic status influences the relationship between diet quality and depressive symptoms.

## 1 Introduction

Depression is a major global public health challenge with substantial consequences for individuals, healthcare systems, and society at large. According to estimates from the World Health Organization, approximately 350 million people worldwide are affected by depression (1), and the burden continues to increase, particularly among adolescents. The COVID-19 pandemic has further intensified this trend, with depressive symptoms in young populations rising markedly during and after the pandemic period (2). Clinically, depression is characterized by persistent low mood, anhedonia, cognitive impairment, and sleep disturbance, and in severe cases may lead to suicidal ideation or behavior. In addition, depression frequently coexists with other psychiatric disorders, such as post-traumatic stress disorder, thereby complicating diagnosis and treatment and reducing therapeutic efficacy (3). At the societal level, depression is associated with increased healthcare utilization, impaired occupational functioning, and reduced quality of life. It is also one of the most common adverse health outcomes among individuals exposed to intimate partner violence, further underscoring its broad psychosocial and economic impact (4). Therefore, identifying modifiable factors associated with depression remains a priority for public health and preventive medicine.

The pathogenesis of depression is multifactorial and involves genetic susceptibility, neurobiological alterations, psychosocial stressors, and lifestyle-related exposures. Among these, diet has emerged as a potentially important and modifiable determinant of mental health (5). Contemporary lifestyles are often accompanied by unhealthy dietary patterns characterized by excessive intake of refined carbohydrates, saturated fat, and energy-dense but micronutrient-poor foods. Such imbalances may contribute to systemic inflammation, oxidative stress, and deficiencies in nutrients essential for normal brain function, thereby increasing vulnerability to depression. In particular, insufficient intake of omega-3 polyunsaturated fatty acids (n-3 PUFAs), which play an important role in neuronal development and neurotransmission, has been linked to a higher risk of depressive symptoms (6). These observations suggest that overall diet quality may be a potentially relevant lifestyle-related factor for depressive symptom burden, although causal effects of dietary improvement cannot be inferred from cross-sectional evidence.

HEI is a comprehensive tool developed to evaluate overall dietary quality according to established dietary guidelines. Rather than focusing on single nutrients or foods, HEI captures adherence to recommended dietary patterns at the population and individual levels. It is based on official dietary recommendations, such as the Dietary Guidelines for Americans issued by the U.S. Department of Agriculture, and has been periodically updated to reflect evolving nutritional evidence and public health priorities (7). Prior studies have demonstrated that HEI has good validity, reliability, and applicability across diverse populations (8, 9). As a multidimensional measure of diet quality, HEI offers an advantage over single-component dietary indicators by better reflecting the complexity of real-world eating behaviors and their potential health effects (10).

To date, a growing body of literature has examined the relationship between dietary patterns and depression. Systematic reviews and meta-analyses have evaluated the effects of different dietary interventions on depressive symptoms, quality of life, and safety outcomes (11). In parallel, increasing attention has been paid to the adverse mental health effects of ultra-processed food consumption (12), as well as to potentially favorable associations of Mediterranean-style dietary patterns, beverage profiles, and specific nutrients or dietary exposures (13, 14). Nevertheless, evidence regarding the association between overall diet quality and depression remains inconsistent, and studies using comprehensive dietary quality indices in large-scale populations remain relatively limited. Moreover, few studies have evaluated whether such associations are reproducible in independent external populations, an important step for strengthening the robustness and generalizability of epidemiological findings.

To address these gaps, the present study examined the cross-sectional association between HEI-2015 and PHQ-9-defined clinically relevant depressive symptoms in a nationally representative U.S. sample from NHANES 2007–March 2020, and then evaluated whether the findings were reproducible in an independent hospital-based replication cohort derived from the Health Examination Center of the Second Affiliated Hospital of Shandong First Medical University. In addition, given the close links between metabolic health, dietary behavior, and mental status, we further examined whether diabetes modified the association between HEI-2015 and depressive symptom outcomes.

## 2 Materials and methods

### 2.1 Study design and sample

**FIGURE 1:**
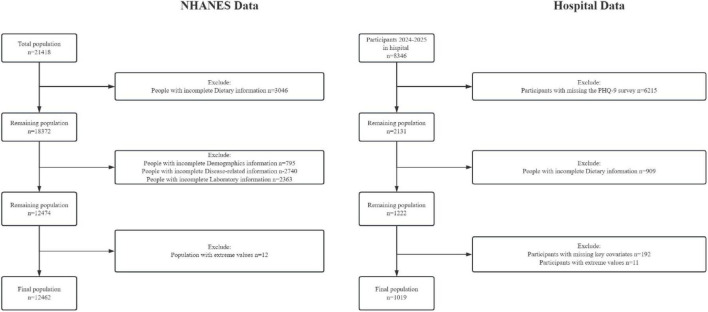
Flowchart for screening the study population. PHQ-9 = 9-item Patient Health Questionnaire. The NHANES initial eligible sample comprised participants with complete PHQ-9 information; the final analytic samples were generated using complete-case exclusion for dietary variables, covariates, and extreme values.

This study was conducted using two independent cross-sectional datasets: a nationally representative U.S. population-based dataset and an independent hospital-based replication cohort. The participant selection procedures for both cohorts are shown in Figure 1. Because the two cohorts differed in sampling frame and dietary assessment, the hospital-based cohort was used to examine reproducibility rather than to provide a strictly identical replication.

For the primary analysis, data were obtained from the National Health and Nutrition Examination Survey (NHANES) 2007–March 2020, a continuous cross-sectional program designed to assess the health and nutritional status of the noninstitutionalized U.S. population. The 2017–March 2020 pre-pandemic file was treated according to the corresponding NHANES analytic guidance. The initial eligible NHANES sample comprised participants with complete PHQ-9 information and eligibility for dietary assessment (*n* = 21,418). After excluding individuals with incomplete dietary information (*n* = 3,046), 18,372 participants remained. We further excluded participants with incomplete demographic information (*n* = 795), incomplete disease-related information (*n* = 2,740), or incomplete laboratory information (*n* = 2,363). After additionally excluding participants with extreme values (*n* = 12), the final analytic sample for the NHANES cohort comprised 12,462 participants. Therefore, all participants included in the final NHANES analysis had complete PHQ-9 data, and a complete-case approach was used for covariates required in the final models.

To examine reproducibility in an independent setting, we established a hospital-based replication cohort consisting of individuals who underwent routine health examinations at the Health Examination Center of the Second Affiliated Hospital of Shandong First Medical University between 2024 and 2025. A total of 8,346 individuals were initially screened. After excluding participants without PHQ-9 data (*n* = 6,215), 2,131 participants remained. We then excluded individuals with incomplete dietary information (*n* = 909), followed by those with missing key covariates (*n* = 192) or extreme values (*n* = 11), yielding a final replication sample of 1,019 participants. Complete-case analysis was used in this cohort as well.

### 2.2 Assessment of depressive symptoms and dietary quality

In both cohorts, depressive symptoms were assessed using the 9-item Patient Health Questionnaire (PHQ-9), a validated self-administered instrument widely used to screen for depressive symptoms in epidemiological studies and clinical practice (15, 16). Each item is scored from 0 to 3, resulting in a total score ranging from 0 to 27. Consistent with established practice, a PHQ-9 score ≥ 10 was used to define clinically relevant depressive symptoms in the present analyses. This outcome should be interpreted as PHQ-9-defined clinically relevant depressive symptoms rather than a physician-confirmed clinical diagnosis of major depressive disorder.

Diet quality was evaluated using HEI-2015, which reflects adherence to the Dietary Guidelines for Americans and ranges from 0 to 100, with higher scores indicating better overall dietary quality. In the NHANES cohort, HEI-2015 was calculated from the first 24-h dietary recall collected through the “What We Eat in America” dietary interview using the Automated Multiple-Pass Method (17, 18). The Day 1 dietary recall was used because it provided the dietary exposure corresponding to the NHANES Day 1 dietary sample weight.

The HEI-2015 score comprises 13 components. The nine adequacy components are total fruits, whole fruits, total vegetables, greens and beans, whole grains, dairy, total protein foods, seafood and plant proteins, and fatty acids. The four moderation components are refined grains, sodium, added sugars, and saturated fats. Component scores were assigned according to established HEI-2015 standards: most food-group components were scored per 1,000 kcal, fatty acids were scored using the ratio of polyunsaturated plus monounsaturated fatty acids to saturated fatty acids, and added sugars and saturated fats were scored as percentages of total energy intake. The maximum total score was 100, with higher scores indicating closer adherence to dietary guidelines (17, 19).

In the hospital-based replication cohort, dietary intake information was collected using a structured health-examination questionnaire that captured major food groups, habitual intake frequency, and beverage-related items available for HEI-2015 scoring. These questionnaire-derived variables were mapped to the same 13 HEI-2015 components as closely as possible and scored using a harmonized framework aligned with the NHANES-based approach. However, the hospital questionnaire was not identical to the NHANES 24-hour dietary recall instrument, and formal cross-cultural validation against the NHANES dietary recall was not available in this dataset. Therefore, the replication-cohort HEI-2015 estimates should be interpreted as closely harmonized but not strictly identical measures.

### 2.3 Covariates

Demographic, socioeconomic, lifestyle, anthropometric, and disease-related covariates were considered as potential confounders.

In the NHANES cohort, covariates included age, sex, race/ethnicity, marital status, family income-to-poverty ratio (PIR), smoking status, alcohol consumption, moderate physical activity duration, BMI, hypertension, diabetes, metabolic syndrome, and cancer. PIR was calculated by NHANES as the ratio of family income to the federal poverty threshold, with higher values indicating higher relative household income. Race/ethnicity was categorized as Mexican American, other Hispanic, non-Hispanic White, non-Hispanic Black, and other race. Marital status was classified as married/living with a partner, widowed/divorced/separated, or never married. Smoking status was categorized as never, former, and current smoking. Alcohol consumption was classified as never, former, mild, moderate, and heavy drinking (20). BMI was calculated as weight in kilograms divided by height in meters squared. Moderate physical activity duration was treated as a continuous variable in minutes as recorded in the physical-activity questionnaire and was dichotomized at 50 min for the prespecified subgroup analyses.

In the hospital-based replication cohort, corresponding covariates included age, sex, marital status, socioeconomic status (SES), smoking status, alcohol consumption, moderate physical activity duration, BMI, hypertension, diabetes, and cancer. Because race/ethnicity and metabolic syndrome data were unavailable or not comparable with the NHANES definitions, these variables were not included in the replication analyses. SES was used as the socioeconomic indicator corresponding to PIR in NHANES. Moderate physical activity duration was recorded in minutes using the hospital health-examination questionnaire and was analyzed in the same manner as in NHANES.

### 2.4 Definition of comorbidities

In the NHANES cohort, diabetes was defined according to any of the following criteria: self-reported physician diagnosis, glycated hemoglobin (HbA1c) > 6.5%, fasting plasma glucose ≥ 7.0 mmol/L, random plasma glucose ≥ 11.1 mmol/L, 2-h oral glucose tolerance test glucose ≥ 11.1 mmol/L, or use of glucose-lowering medication or insulin (21). Metabolic syndrome was defined based on the National Cholesterol Education Program Adult Treatment Panel III criteria, requiring the presence of at least three of the following components: elevated fasting glucose or glucose-lowering treatment, reduced high-density lipoprotein cholesterol, elevated triglycerides or lipid-lowering treatment, increased waist circumference, and elevated blood pressure or antihypertensive treatment (22). Hypertension was defined as an average blood pressure ≥ 140/90 mmHg. Cancer history was determined from self-reported physician diagnosis. Before analysis, the diabetes variable was verified so that “Yes” denoted participants meeting the diabetes definition and “No” denoted participants not meeting the definition.

In the hospital-based replication cohort, hypertension, diabetes, and cancer were defined using the corresponding clinical examination, laboratory, medication, and questionnaire data available from the hospital-based health examination system. The diabetes coding was checked in the same direction as in NHANES, with “Yes” indicating diabetes and “No” indicating no diabetes. Metabolic syndrome was not included because the corresponding data were not available in a form fully comparable to the NHANES definition.

### 2.5 Statistical analyses

All statistical analyses were performed using R software (version 4.2.1). Continuous variables are presented as means with standard deviations or standard errors as appropriate, and categorical variables are presented as frequencies and percentages. Normality of continuous variables, including PHQ-9 score, was assessed using distributional plots and the D’Agostino–Pearson normality test. As expected for symptom scores, PHQ-9 was right-skewed in both cohorts (both *P* < 0.001); therefore, analyses using PHQ-9 score were interpreted as adjusted mean differences in symptom burden rather than as evidence of normally distributed symptom severity. Differences between groups were compared using Student’s *t*-test or survey-weighted linear tests for continuous variables and the chi-square test or survey-weighted chi-square test for categorical variables, as appropriate.

To evaluate the association between HEI and PHQ-9-defined clinically relevant depressive symptoms, multivariable logistic regression models were used with clinically relevant depressive symptoms defined as PHQ-9 ≥ 10. In addition, multivariable linear regression models were fitted using PHQ-9 score as a continuous outcome to assess the association between HEI and depressive symptom burden. In the logistic models, odds ratios (ORs) were interpreted per 1-point increase in HEI-2015, and clinically interpretable estimates per 5-point and 10-point increase were additionally derived from the model coefficients.

For the NHANES cohort, three progressively adjusted models were constructed. Model 1 adjusted for age, sex, and race/ethnicity. Model 2 further adjusted for marital status, moderate physical activity duration, BMI, PIR, smoking status, and alcohol consumption. Model 3 additionally adjusted for hypertension, diabetes, metabolic syndrome, and cancer. Covariates were selected a priori based on prior literature, clinical relevance, and their potential associations with both diet quality and depressive symptoms. Because diabetes was the key effect modifier, interaction models included both the main effect of diabetes and a HEI × diabetes product term; in diabetes-stratified analyses, diabetes itself was not adjusted within each stratum.

For the hospital-based replication cohort, the same analytical framework was applied with modifications based on variable availability. Specifically, Model 1 adjusted for age and sex; Model 2 further adjusted for marital status, moderate physical activity duration, BMI, SES, smoking status, and alcohol consumption; and Model 3 additionally adjusted for hypertension, diabetes, and cancer. Race/ethnicity and metabolic syndrome were not included in the replication models because comparable variables were not available.

Subgroup analyses were performed to assess potential effect modification. In the NHANES cohort, stratified analyses were conducted according to age, sex, race/ethnicity, marital status, PIR, BMI, moderate physical activity duration, smoking status, alcohol consumption, hypertension, metabolic syndrome, diabetes, and cancer. In the hospital-based replication cohort, subgroup analyses were conducted using the same strategy except that race/ethnicity and metabolic syndrome were omitted and PIR was replaced by SES. Interaction terms were used to test for heterogeneity across subgroups. Because multiple subgroup tests were performed, these analyses were considered exploratory and hypothesis-generating, with emphasis placed on interactions that were directionally consistent and reproducible across both cohorts.

Restricted cubic spline models with three knots located at the 10th, 50th, and 90th percentiles of HEI were further fitted to examine the dose-response association between HEI and clinically relevant depressive symptoms and to assess potential nonlinearity in both cohorts. The median HEI value was used as the reference. The revised spline figures show the reference value, knot locations, confidence intervals, and the distribution of HEI values.

For NHANES, all analyses accounted for the complex multistage sampling design, including strata, primary sampling units, and dietary sample weights. Because HEI-2015 was derived from the first 24-h dietary recall, Day 1 dietary sample weights were used. For combined cycles, dietary weights were rescaled across the included NHANES cycles, and the 2017–March 2020 pre-pandemic dietary weight was handled according to NHANES analytic guidance before combination with earlier cycles. The complex sampling design was incorporated into logistic regression, linear regression, subgroup analyses, and restricted cubic spline analyses. Because the hospital-based replication cohort was not population-based, no survey weighting was applied in that cohort. All tests were two-sided, and *P* < 0.05 was considered statistically significant.

Model diagnostics were added in response to the reviewer’s comment. Collinearity was assessed using variance inflation factors (VIFs) in the fully adjusted model. The maximum VIF was 3.13 in NHANES and 4.19 in the replication cohort, and the VIFs for diabetes, hypertension, and metabolic syndrome were all below 1.50 in NHANES, suggesting that severe multicollinearity was unlikely. As a sensitivity analysis for the diabetes interaction in NHANES, models excluding metabolic syndrome were also examined; the direction of the diabetes-specific HEI associations and the HEI × diabetes interaction were materially unchanged.

## 3 Results

### 3.1 Characteristics of the included population

Baseline characteristics stratified by depression status are shown in Tables 1, 2. In the NHANES cohort, participants with depression were younger, more likely to be female, and more frequently unmarried, divorced, or separated than those without depression (all *P* < 0.001). They also had higher BMI, lower PIR, and a less favorable behavioral profile, including a higher prevalence of current smoking and heavy alcohol consumption (all *P* < 0.001). In addition, hypertension, metabolic syndrome, and diabetes were more common among participants with depression. Notably, depressed participants had significantly lower HEI-2015 scores than non-depressed participants (*P* < 0.001), and inadequate diet quality was more prevalent in the depressed group.

**TABLE 1:** Baseline characteristics of the population in the NHANES cohort.

Variable	Depression			
	Total	No	Yes	*P*-value
Total	12,462 (100.000)	11,728 (94.618)	734 (5.382)	
Age∼years	46.068 (0.295)	46.255 (0.308)	42.789 (0.797)	< 0.001
Sex∼%				< 0.001
Male	6,251 (48.895)	5,968 (49.454)	283 (39.066)	
Female	6,211 (51.105)	5,760 (50.546)	451 (60.934)	
Race∼%				0.004
Non-Hispanic White	5,789 (73.150)	5,464 (73.390)	325 (68.930)	
Non-Hispanic Black	2,449 (8.609)	2,296 (8.526)	153 (10.070)	
Mexican American	1,454 (6.226)	1,363 (6.211)	91 (6.488)	
Other Hispanic	1,075 (4.606)	985 (4.450)	90 (7.353)	
Other Race	1,695 (7.408)	1,620 (7.422)	75 (7.160)	
Marital status				< 0.001
Married/Living with partner	7,520 (65.014)	7,201 (66.089)	319 (46.115)	
Widowed/Divorced/Separated	2,422 (15.943)	2,197 (15.389)	225 (25.688)	
Never married	2,520 (19.043)	2,330 (18.522)	190 (28.197)	
BMI∼kg/m^2^	28.449 (0.099)	28.337 (0.097)	30.435 (0.389)	< 0.001
Family PIR	3.409 (0.037)	3.453 (0.037)	2.630 (0.095)	< 0.001
Smoking behavior∼%				< 0.001
Never	7,360 (59.284)	7,030 (60.006)	330 (46.602)	
Former	3,069 (25.916)	2,894 (25.974)	175 (24.884)	
Now	2,033 (14.800)	1,804 (14.020)	229 (28.514)	
Alcohol consumption∼%				< 0.001
Never	1,350 (8.376)	1,277 (8.444)	73 (7.188)	
Former	1,257 (8.184)	1,161 (7.933)	96 (12.584)	
Mild	5,032 (41.556)	4,821 (42.216)	211 (29.942)	
Moderate	2,293 (20.179)	2,150 (20.097)	143 (21.619)	
Heavy	2,530 (21.705)	2,319 (21.309)	211 (28.666)	
Moderate exercise∼minutes	64.908(0.772)	65.100(0.802)	61.531 (3.076)	0.267
Hypertension				0.013
No	7,713 (67.014)	7,299 (67.375)	414 (60.657)	
Yes	4,749 (32.986)	4,429 (32.625)	320 (39.343)	
Metabolic syndrome∼%				0.003
No	9,756 (80.728)	9,239 (81.067)	517 (74.769)	
Yes	2,706 (19.272)	2,489 (18.933)	217 (25.231)	
Diabetes∼%				0.030
No	10,615(89.251)	10,024 (89.458)	591 (85.614)	
Yes	18,47(10.749)	1,704 (10.542)	143 (14.386)	
Cancer∼%				0.709
No	11,298 (89.696)	10,630 (89.665)	668 (90.238)	
Yes	1,164(10.304)	1,098(10.335)	66 (9.762)	
HEI	52.971 (0.261)	53.172 (0.265)	49.435 (0.728)	< 0.001
Inadequate	5,417 (43.862)	5,025 (43.366)	392 (52.585)	
Average	5,410 (43.127)	5,140 (43.493)	270 (36.702)	
Optimal	1,635 (13.010)	1,563 (13.141)	72 (10.713)	

Data are presented as weighted means (standard errors) or unweighted counts (weighted percentages) for NHANES. PHQ-9-defined clinically relevant depressive symptoms were defined as PHQ-9 ≥ 10. BMI, body mass index; PIR, family income-to-poverty ratio; HEI, Healthy Eating Index-2015; MetS, metabolic syndrome. Diabetes “Yes” indicates participants meeting the diabetes definition; “No” indicates participants not meeting the definition.

**TABLE 2:** Baseline characteristics of the population in the hospital-based replication cohort.

Variable	Depression			
	Total	No	Yes	*P*-value
Total	1019 (100.000)	961 (94.308)	58 (5.692)	
Age∼years	50.070 (11.954)	50.078 (11.934)	49.931 (12.400)	0.930
Sex∼%				0.877
Male	561 (55.054)	528 (54.943)	33 (56.897)	
Female	458 (44.946)	433 (45.057)	25 (43.103)	
Marital Status				0.136
Married/Living with Partner	790 (77.527)	751 (78.148)	39 (67.241)	
Widowed/Divorced/Separated	110 (10.795)	100 (10.406)	10 (17.241)	
Never married	119 (11.678)	110 (11.446)	9 (15.517)	
BMI∼kg/m^2^	26.323 (3.001)	26.263 (2.998)	27.310 (2.911)	0.010
SES	3.186 (0.806)	3.193 (0.806)	3.073 (0.809)	0.276
Smoking behavior∼%				0.010
Never	674 (66.143)	644 (67.014)	30 (51.724)	
Former	161 (15.800)	152 (15.817)	9 (15.517)	
Now	184 (18.057)	165 (17.170)	19 (32.759)	
Alcohol consumption∼%				0.487
Never	298 (29.244)	277 (28.824)	21 (36.207)	
Former	69 (6.771)	64 (6.660)	5 (8.621)	
Mild	394 (38.665)	376 (39.126)	18 (31.034)	
Moderate	147 (14.426)	141 (14.672)	6 (10.345)	
Heavy	111 (10.893)	103 (10.718)	8 (13.793)	
Moderate exercise∼minutes	89.446 (37.790)	89.813 37.932)	83.362 (35.099)	0.181
Hypertension				0.876
No	651 (63.886)	615 (63.996)	36 (62.069)	
Yes	368 (36.114)	346 (36.004)	22 (37.931)	
Diabetes				0.020
No	843 (82.728)	802 (83.455)	41 (70.690)	
Yes	176 (17.272)	159 (16.545)	17 (29.310)	
Cancer∼%				0.716
No	852 (83.611)	805 (83.767)	47 (81.034)	
Yes	167 (16.389)	156 (16.233)	11 (18.966)	
HEI	57.566 (7.426)	57.725 (7.351)	54.923 (8.201)	0.014
Inadequate	340 (33.366)	307 (31.946)	33 (56.897)	< 0.001
Average	339 (33.268)	328 (34.131)	11 (18.966)	
Optimal	340(33.366)	326 (33.923)	14 (24.138)	

Data are presented as means (standard deviations) or counts (percentages) for the hospital-based replication cohort. PHQ-9-defined clinically relevant depressive symptoms were defined as PHQ-9 ≥ 10. BMI, body mass index; SES, socioeconomic status; HEI, Healthy Eating Index-2015.

The hospital-based replication cohort showed a concordant pattern. Compared with non-depressed participants, those with depression had higher BMI (*P* = 0.010), were more likely to be current smokers (*P* = 0.010), and had a higher prevalence of diabetes (*P* = 0.020). HEI scores were also significantly lower among depressed individuals (*P* = 0.014). Consistently, inadequate diet quality was markedly overrepresented in the depression group (*P* < 0.001). Other variables, including age, sex, marital status, SES, alcohol intake, physical activity, hypertension, and cancer, did not differ significantly by depression status in the replication cohort. Overall, both datasets demonstrated that depression clustered with poorer dietary quality and a less favorable metabolic-behavioral profile.

### 3.2 Association between HEI and PHQ-9-defined clinically relevant depressive symptoms

As shown in Table 3, higher HEI was associated with lower odds of PHQ-9-defined clinically relevant depressive symptoms and lower PHQ-9 score in both datasets. In NHANES, this inverse association remained statistically significant after full adjustment for demographic factors, lifestyle variables, and comorbidities (Model 3: OR per 1-point increase = 0.992, 95% CI: 0.984–1.000, *P* = 0.048). This corresponds to approximately 0.8% lower odds per 1-point higher HEI and approximately 7.7% lower odds per 10-point higher HEI (OR per 10-point increase = 0.923). Higher HEI was also associated with lower PHQ-9 scores in the fully adjusted model (β per 1-point increase = −0.008, 95% CI: −0.014 to −0.002, *P* = 0.006), corresponding to an adjusted difference of approximately −0.08 PHQ-9 points per 10-point higher HEI.

**TABLE 3:** Association between HEI and PHQ-9-defined clinically relevant depressive symptoms and PHQ-9 score.

Data	Model	Depression		PHQ-9 Score	
		OR (95%CI)	*P*-value	β (95%CI)	*P*-value
NHANES data	Crude	0.981 (0.974, 0.988)	< 0.001	−0.022 (−0.027, −0.017)	<0.001
	Model 1	0.982 (0.975, 0.989)	< 0.001	−0.021 (−0.026, −0.015)	<0.001
	Model 2	0.991(0.983, 0.999)	0.035	−0.009 (−0.014, −0.003)	0.003
	Model 3	0.992(0.984, 1.000)	0.048	−0.008 (−0.014, −0.002)	0.006
Replication data	Crude	0.951(0.917, 0.985)	0.005	−0.065 (−0.092, −0.038)	<0.001
	Model 1	0.949(0.914, 0.984)	0.005	−0.069 (−0.097, −0.041)	<0.001
	Model 2	0.959(0.923, 0.996)	0.028	−0.053 (−0.080, −0.025)	<0.001
	Model 3	0.960(0.925, 0.997)	0.036	−0.049 (−0.077, −0.022)	<0.001

ORs and β coefficients are reported per 1-point increase in HEI-2015. The binary outcome denotes PHQ-9-defined clinically relevant depressive symptoms (PHQ-9 ≥ 10), whereas PHQ-9 score is a continuous measure of depressive symptom burden. Model 1 adjusted for age, sex, and race/ethnicity in NHANES and for age and sex in the replication cohort. Model 2 additionally adjusted for marital status, moderate physical activity duration, BMI, socioeconomic indicators, smoking, and alcohol consumption. Model 3 additionally adjusted for comorbidities available in each cohort.

These findings were reproduced in the hospital-based replication cohort. In the fully adjusted model, each 1-point increase in HEI was associated with lower odds of PHQ-9-defined clinically relevant depressive symptoms (OR = 0.960, 95% CI: 0.925–0.997, *P* = 0.036), corresponding to approximately 4.0% lower odds per 1-point higher HEI and approximately 33.5% lower odds per 10-point higher HEI (OR per 10-point increase = 0.665). Higher HEI was also associated with lower PHQ-9 score (β = −0.049, 95% CI: −0.077 to −0.022, *P* < 0.001), corresponding to an adjusted difference of approximately −0.49 PHQ-9 points per 10-point higher HEI. Thus, the binary outcome reflects PHQ-9-defined clinically relevant depressive symptoms, whereas the continuous PHQ-9 score reflects overall depressive symptom burden.

### 3.3 Subgroup analysis of HEI and PHQ-9-defined clinically relevant depressive symptoms

Subgroup analyses showed that the inverse association between HEI and PHQ-9-defined clinically relevant depressive symptoms was generally similar across most strata. However, diabetes status significantly modified this association in both datasets. In NHANES, higher HEI was associated with lower odds of clinically relevant depressive symptoms among participants without diabetes (OR per 1-point increase = 0.987, 95% CI: 0.978–0.996, *P* = 0.004; OR per 10-point increase = 0.877), whereas the association was directionally opposite among those with diabetes (OR = 1.024, 95% CI: 1.006–1.042, *P* = 0.011; OR per 10-point increase = 1.268; *P* for interaction < 0.001) (Table 4). Because these are cross-sectional associations, the opposite direction among participants with diabetes should not be interpreted as evidence that a healthier diet causes depressive symptoms.

**TABLE 4:** Results of subgroup analyses of HEI and odds of clinically relevant depressive symptoms in the NHANES cohort.

Subgroup variable	Depression		
	OR (95%CI)	*P*-value	*P* for interaction
Age			0.364
< 65	0.991 (0.982,0.999)	0.033	
≥ 65	0.998 (0.976, 1.020)	0.843	
Sex			0.489
Female	0.997 (0.988,1.007)	0.558	
Male	0.983 (0.968,0.999)	0.033	
Race			0.143
Mexican American	0.989 (0.972,1.007)	0.218	
Non-Hispanic Black	0.980 (0.961,1.000)	0.046	
Non-Hispanic White	0.990 (0.979,1.000)	0.054	
Other Hispanic	0.999 (0.978,1.020)	0.924	
Other Race	1.017 (0.994, 1.041)	0.142	
Marital status			0.432
Married/living with partner	0.998 (0.984, 1.012)	0.799	
Never married	0.983 (0.968, 0.997)	0.02	
Widowed/divorced/separated	0.990 (0.976, 1.004)	0.152	
Family PIR			0.386
≤ 1.3	0.995 (0.986, 1.005)	0.321	
1.3–3.5	0.994 (0.981, 1.007)	0.333	
> 3.5	0.987 (0.970, 1.005)	0.153	
BMI			0.083
< 30	0.988 (0.978, 0.998)	0.021	
≥ 30	0.998 (0.984, 1.012)	0.803	
Moderate exercise			0.168
< 50	0.986 (0.972, 1.000)	0.044	
≥ 50	0.999 (0.988, 1.011)	0.889	
Smoking behaviour			0.448
Former	0.995 (0.976, 1.015)	0.621	
Never	0.993 (0.980, 1.005)	0.244	
Now	0.985 (0.973, 0.997)	0.014	
Alcohol consumption∼%			0.756
Never	0.991 (0.968, 1.014)	0.418	
Former	0.998 (0.975, 1.020)	0.837	
Mild	0.992 (0.974, 1.011)	0.395	
Moderate	0.981 (0.966, 0.997)	0.023	
Heavy	0.997 (0.983, 1.012)	0.689	
Hypertension			0.633
No	0.990 (0.980, 1.000)	0.061	
Yes	0.995 (0.982, 1.009)	0.475	
Metabolic syndrome			0.056
No	0.986 (0.976, 0.996)	0.009	
Yes	1.009 (0.992, 1.025)	0.301	
Diabetes			< 0.001
No	0.987 (0.978, 0.996)	0.004	
Yes	1.024 (1.006, 1.042)	0.011	
Cancer			0.167
No	0.989 (0.981, 0.998)	0.016	
Yes	1.012 (0.985, 1.040)	0.376	

ORs are reported per 1-point increase in HEI-2015. Subgroup models were adjusted according to Model 3, except that the stratifying variable was not adjusted within its own subgroup. PHQ-9-defined clinically relevant depressive symptoms were defined as PHQ-9 ≥ 10. PIR, family income-to-poverty ratio; BMI, body mass index; HEI, Healthy Eating Index-2015.

This interaction pattern was reproduced in the hospital-based replication cohort. Among individuals without diabetes, higher HEI was associated with lower odds of PHQ-9-defined clinically relevant depressive symptoms (OR = 0.902, 95% CI: 0.859–0.947, *P* < 0.001; OR per 10-point increase = 0.357), whereas among those with diabetes, higher HEI was associated with higher odds (OR = 1.121, 95% CI: 1.022–1.228, *P* = 0.015; OR per 10-point increase = 3.134; *P* for interaction < 0.001). These results identify diabetes as the most reproducible effect modifier of the HEI–depressive symptom association, although the smaller diabetic subgroup and potential post-diagnosis dietary changes warrant cautious interpretation (Table 5).

**TABLE 5:** Results of subgroup analyses of HEI and odds of clinically relevant depressive symptoms in the hospital-based replication cohort.

Subgroup variable	Depression		
	OR (95%CI)	*P*-value	*P* for interaction
Age			0.815
< 65	0.959 (0.921, 0.999)	0.046	
≥ 65	0.909 (0.806, 1.026)	0.122	
Sex			0.561
Female	0.941 (0.881, 1.006)	0.074	
Male	0.971 (0.925, 1.020)	0.238	
Marital Status			0.395
Married/Living with Partner	0.956 (0.912, 1.002)	0.059	
Never married	0.975 (0.867, 1.097)	0.676	
Widowed/Divorced/Separated	0.929 (0.837, 1.031)	0.165	
SES			0.193
≤ 2.5	0.876 (0.797, 0.964)	0.007	
2.5–3.5	0.985 (0.930, 1.044)	0.61	
> 3.5	0.963 (0.890, 1.041)	0.338	
BMI			0.476
< 30	0.947 (0.905, 0.991)	0.018	
≥ 30	0.966 (0.871, 1.072)	0.52	
Moderate exercise			0.439
< 50	0.747 (0.575, 0.969)	0.028	
≥ 50	0.962 (0.924, 1.001)	0.059	
Smoking behavior			0.764
Former	0.975 (0.873, 1.090)	0.659	
Never	0.958 (0.906, 1.013)	0.132	
Now	0.950 (0.887, 1.016)	0.135	
Alcohol consumption			0.158
Never	0.974 (0.911, 1.040)	0.428	
Former	0.975 (0.748, 1.272)	0.851	
Mild	0.964 (0.901, 1.032)	0.292	
Moderate	0.707 (0.544, 0.919)	0.01	
Heavy	0.933 (0.787, 1.106)	0.422	
Hypertension			0.095
No	0.930 (0.885, 0.978)	0.004	
Yes	1.014 (0.951, 1.081)	0.675	
Diabetes			< 0.001
No	0.902 (0.859, 0.947)	< 0.001	
Yes	1.121 (1.022, 1.228)	0.015	
Cancer			0.796
No	0.966 (0.926, 1.007)	0.104	
Yes	0.941 (0.843, 1.052)	0.285	

ORs are reported per 1-point increase in HEI-2015. Subgroup models were adjusted according to Model 3, except that the stratifying variable was not adjusted within its own subgroup. PHQ-9-defined clinically relevant depressive symptoms were defined as PHQ-9 ≥ 10. SES, socioeconomic status; BMI, body mass index; HEI, Healthy Eating Index-2015.

### 3.4 Subgroup analysis of HEI and PHQ-9 score

A similar pattern was observed for depressive symptom burden. In the NHANES cohort, higher HEI was generally associated with lower PHQ-9 scores, although the strength and direction of this association varied across subgroups. Significant interactions were observed for BMI (P for interaction = 0.042), smoking behavior (P for interaction = 0.011), metabolic syndrome (P for interaction = 0.033), and diabetes (P for interaction = 0.012). Specifically, an inverse association was observed among participants with BMI < 30 kg/m^2^ (β = −0.012, 95% CI: −0.019 to −0.006, *P* < 0.001), whereas no significant association was found among those with BMI ≥ 30 kg/m^2^ (β = 0.000, 95% CI: −0.011 to 0.011, *P* = 0.982). For smoking behavior, the inverse association was most evident among current smokers (β = −0.028, 95% CI: −0.044 to −0.012, *P* < 0.001), but was not statistically significant among former or never smokers. For metabolic syndrome, higher HEI was significantly associated with lower PHQ-9 scores among participants with metabolic syndrome (β = −0.012, 95% CI: −0.019 to −0.005, *P* < 0.001), whereas no significant association was observed among those without metabolic syndrome (β = 0.008, 95% CI: −0.005 to 0.021, *P* = 0.217). Most notably, diabetes significantly modified this association: higher HEI was associated with lower PHQ-9 scores among participants without diabetes (β = −0.011, 95% CI: −0.017 to −0.005, *P* < 0.001; approximately −0.11 PHQ-9 points per 10-point higher HEI), but with higher PHQ-9 scores among those with diabetes (β = 0.018, 95% CI: 0.001–0.035, *P* = 0.038; approximately 0.18 PHQ-9 points per 10-point higher HEI) (Table 6).

**TABLE 6:** Results of subgroup analyses of HEI and PHQ-9 score in the NHANES cohort.

Subgroup variable	PHQ-9 Score		
	β (95%CI)	*P-*value	*P* for interaction
Age			0.243
< 65	−0.009 (−0.016, −0.003)	0.005	
≥ 65	−0.006 (−0.015, 0.004)	0.243	
Sex			0.127
Female	−0.008 (−0.016, 0.000)	0.045	
Male	−0.008 (−0.017, 0.001)	0.078	
Race			0.738
Mexican American	−0.014 (−0.031, 0.003)	0.113	
Non-Hispanic Black	−0.015 (−0.030, −0.001)	0.036	
Non-Hispanic White	−0.007 (−0.014, 0.000)	0.042	
Other Hispanic	−0.008 (−0.033, 0.017)	0.507	
Other Race	−0.003 (−0.017, 0.011)	0.702	
Marital Status			0.102
Married/Living with Partner	−0.005 (−0.012, 0.002)	0.143	
Never married	−0.017 (−0.030, −0.004)	0.009	
Widowed/Divorced/Separated	−0.008 (−0.025, 0.009)	0.348	
Family PIR			0.779
≤ 1.3	−0.013 (−0.030, 0.004)	0.131	
1.3–3.5	−0.010 (−0.020, 0.000)	0.062	
> 3.5	−0.007 (−0.014, 0.001)	0.068	
BMI			0.042
< 30	−0.012 (−0.019, −0.006)	< 0.001	
≥ 30	0.000 (−0.011, 0.011)	0.982	
Moderate exercise			0.341
< 50	−0.010 (−0.020, −0.001)	0.037	
≥ 50	−0.005 (−0.012, 0.003)	0.219	
Smoking behavior			0.011
Former	−0.002 (−0.013, 0.008)	0.667	
Never	−0.006 (−0.013, 0.001)	0.091	
Now	−0.028 (−0.044, −0.012)	< 0.001	
Alcohol consumption∼%			0.183
Never	0.001 (−0.012, 0.013)	0.932	
Former	−0.029 (−0.054, −0.004)	0.022	
Mild	−0.001 (−0.010, 0.008)	0.829	
Moderate	−0.016 (−0.030, −0.001)	0.031	
Heavy	−0.011 (−0.025, 0.004)	0.139	
Hypertension			0.464
No	−0.009 (−0.016, −0.001)	0.02	
Yes	−0.006 (−0.015, 0.003)	0.181	
Metabolic syndrome			0.033
No	0.008 (−0.005, 0.021)	0.217	
Yes	−0.012 (−0.019, −0.005)	< 0.001	
Diabetes			0.012
No	−0.011 (−0.017, −0.005)	< 0.001	
Yes	0.018 (0.001, 0.035)	0.038	
Cancer			0.317
No	−0.010 (−0.016, −0.003)	0.003	
Yes	0.004 (−0.018, 0.025)	0.742	

β coefficients represent the adjusted difference in PHQ-9 score per 1-point increase in HEI-2015. Subgroup models were adjusted according to Model 3, except that the stratifying variable was not adjusted within its own subgroup. PIR, family income-to-poverty ratio; BMI, body mass index; HEI, Healthy Eating Index-2015.

In the hospital-based replication cohort, diabetes remained the only significant effect modifier. Higher HEI was associated with lower PHQ-9 scores among non-diabetic individuals (β = −0.111, 95% CI: −0.139 to −0.082, *P* < 0.001; approximately −1.11 PHQ-9 points per 10-point higher HEI), whereas a positive association was observed among diabetic individuals (β = 0.234, 95% CI: 0.163–0.306, *P* < 0.001; approximately 2.34 PHQ-9 points per 10-point higher HEI), with a significant interaction (P for interaction < 0.001). Therefore, although several subgroup interactions were identified in the NHANES analysis, the modifying effect of diabetes was the most reproducible finding and was consistently observed in the replication cohort (Table 7).

**TABLE 7:** Results of subgroup analyses of HEI and PHQ-9 score in the hospital-based replication cohort.

Subgroup variable	PHQ-9 Score		
	β (95%CI)	*P*-value	*P* for interaction
Age			0.922
< 65	−0.051 (−0.080, −0.022)	< 0.001	
≥ 65	−0.070 (−0.169, 0.028)	0.165	
Sex			0.446
Female	−0.060 (−0.102, −0.017)	0.007	
Male	−0.038 (−0.075, −0.001)	0.045	
Marital status			0.815
Married/living with partner	−0.047 (−0.078, −0.015)	0.004	
Never married	−0.065 (−0.155, 0.025)	0.159	
Widowed/divorced/separated	−0.048 (−0.140, 0.043)	0.304	
SES			0.319
≤ 2.5	−0.079 (−0.148, −0.009)	0.027	
2.5–3.5	−0.033 (−0.075, 0.009)	0.121	
> 3.5	−0.048 (−0.094, −0.003)	0.036	
BMI			0.143
< 30	−0.058 (−0.087, −0.028)	< 0.001	
≥ 30	0.003 (−0.089, 0.096)	0.944	
Moderate exercise			0.692
< 50	−0.053 (−0.144, 0.039)	0.264	
≥ 50	−0.052 (−0.082, −0.023)	< 0.001	
Smoking behavior			0.798
Former	−0.036 (−0.111, 0.039)	0.352	
Never	−0.054 (−0.088, −0.020)	0.002	
Now	−0.054 (−0.126, 0.018)	0.142	
Alcohol consumption			0.933
never	−0.033 (−0.090, 0.023)	0.246	
Former	−0.084 (−0.206, 0.038)	0.183	
Mild	−0.053 (−0.095, −0.011)	0.013	
Moderate	−0.071 (−0.139, −0.004)	0.04	
Heavy	−0.044 (−0.151, 0.063)	0.421	
Hypertension			0.09
No	−0.072 (−0.105, −0.039)	< 0.001	
Yes	−0.008 (−0.058, 0.043)	0.762	
Diabetes			< 0.001
No	−0.111 (−0.139, −0.082)	< 0.001	
Yes	0.234 (0.163, 0.306)	< 0.001	
Cancer			0.895
No	−0.045 (−0.075, −0.014)	0.004	
Yes	−0.060 (−0.131, 0.011)	0.101	

β coefficients represent the adjusted difference in PHQ-9 score per 1-point increase in HEI-2015. Subgroup models were adjusted according to Model 3, except that the stratifying variable was not adjusted within its own subgroup. SES, socioeconomic status; BMI, body mass index; HEI, Healthy Eating Index-2015.

### 3.5 Nonlinear relationship between HEI and clinically relevant depressive symptoms

**FIGURE 2:**
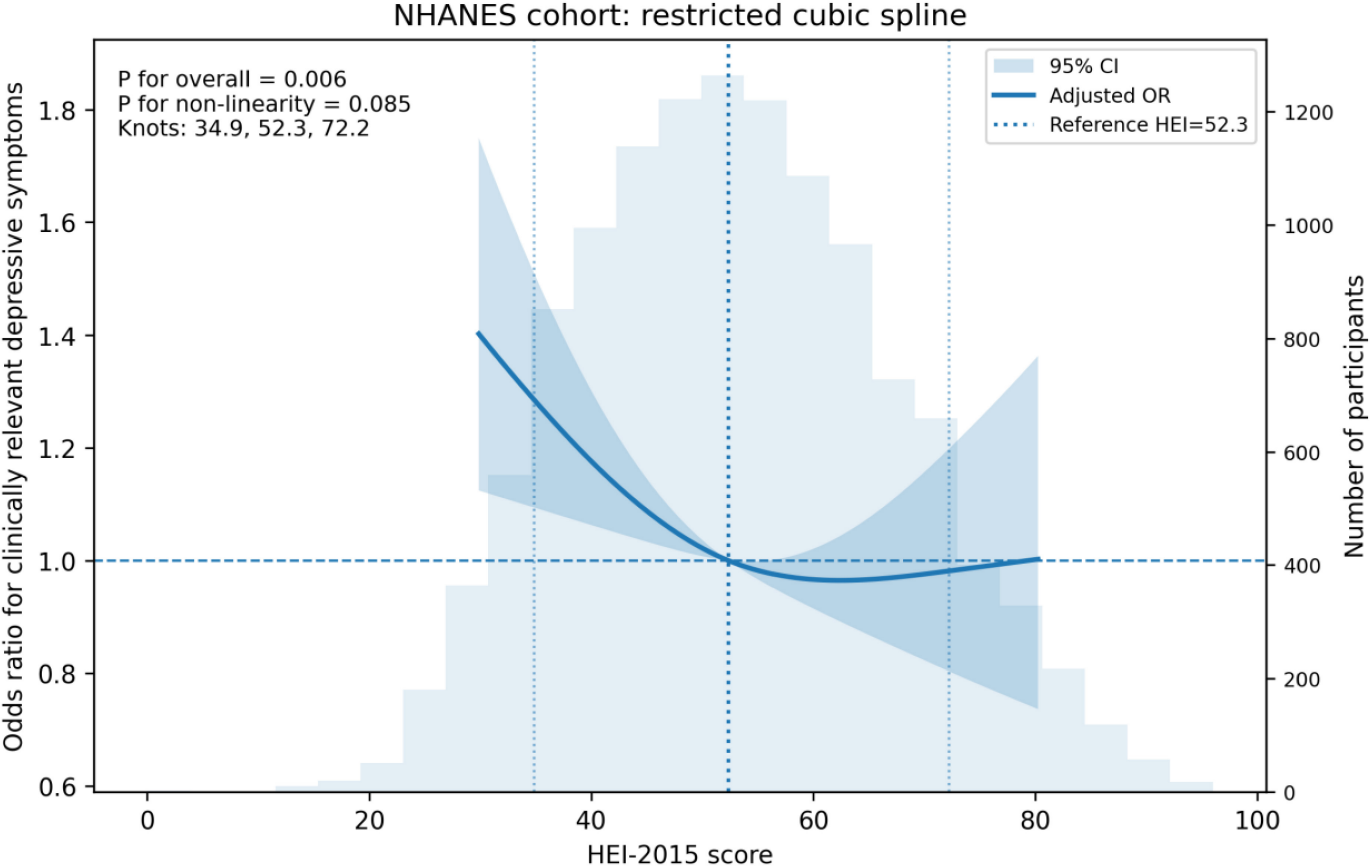
Restricted cubic spline showing the association between HEI and PHQ-9-defined clinically relevant depressive symptoms in the NHANES cohort. The median HEI was used as the reference, vertical dotted lines indicate knot locations, the shaded band indicates the 95% CI, and the background histogram shows the number of observations across the HEI distribution.

Restricted cubic spline analyses were performed to further explore the dose-response relationship between HEI and PHQ-9-defined clinically relevant depressive symptoms. In the NHANES cohort, HEI was significantly associated with clinically relevant depressive symptoms overall (P for overall = 0.006), whereas the test for non-linearity was not statistically significant (P for non-linearity = 0.085). The revised spline plot uses the median HEI as the reference value and displays the knot locations, 95% confidence interval, and HEI distribution. The curve suggested that the odds of clinically relevant depressive symptoms decreased with increasing HEI at lower-to-moderate score ranges and then tended to plateau at higher HEI levels (Figure 2).

**FIGURE 3:**
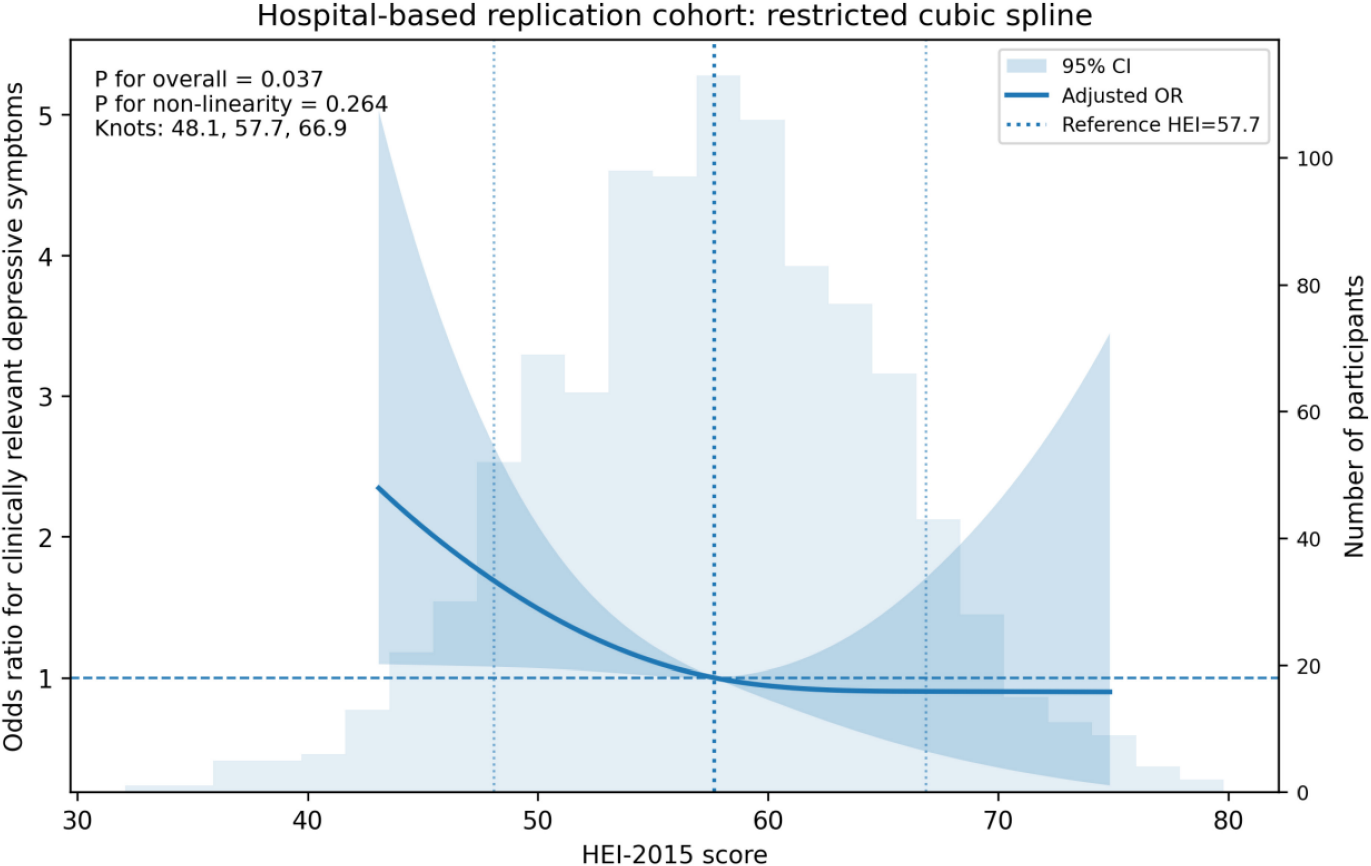
Restricted cubic spline showing the association between HEI and PHQ-9-defined clinically relevant depressive symptoms in the hospital-based replication cohort. The median HEI was used as the reference, vertical dotted lines indicate knot locations, the shaded band indicates the 95% CI, and the background histogram shows the number of observations across the HEI distribution. The y-axis was corrected to display odds ratios rather than log-odds values.

A similar pattern was observed in the hospital-based replication cohort. The overall association between HEI and PHQ-9-defined clinically relevant depressive symptoms remained statistically significant (*P* for overall = 0.037), but there was no evidence of a significant non-linear relationship (*P* for non-linearity = 0.264). The revised curve shows a generally decreasing trend in the odds of clinically relevant depressive symptoms with increasing HEI, with a less pronounced change at higher HEI levels (Figure 3).

## 4 Discussion

In this study, using a nationally representative NHANES cohort and an independent hospital-based replication cohort, we found that higher HEI-2015 scores were cross-sectionally associated with lower odds of PHQ-9-defined clinically relevant depressive symptoms and lower depressive symptom burden. This association remained statistically significant after adjustment for a broad range of demographic, lifestyle, and clinical covariates. Importantly, the main findings were reproduced in the hospital-based cohort, supporting the consistency of the observed association across two independent datasets while acknowledging differences in sampling and dietary assessment. Another notable finding was that diabetes consistently modified this relationship: among individuals without diabetes, higher HEI was associated with more favorable depression-related outcomes, whereas among those with diabetes, the association was directionally opposite. In addition, restricted cubic spline analyses supported a significant overall association between HEI and clinically relevant depressive symptoms in both cohorts, without strong evidence of statistically significant nonlinearity.

A key finding of the present study was that diabetes acted as a reproducible effect modifier. In both the NHANES and hospital-based replication cohorts, higher HEI was associated with more favorable depression-related outcomes among non-diabetic individuals, but the association was directionally opposite among those with diabetes. This pattern was observed not only for PHQ-9-defined clinically relevant depressive symptoms but also for continuous PHQ-9 scores, suggesting that the interaction is unlikely to be fully explained by the choice of outcome definition. Nevertheless, the mechanisms underlying this opposite-direction association remain uncertain, and causal interpretation is not warranted. Individuals with diabetes often face long-term dietary restrictions and intensive self-management demands, which may increase psychological burden and reduce dietary satisfaction. In addition, diabetes is characterized by insulin resistance, chronic low-grade inflammation, and impaired metabolic flexibility, all of which may alter the relationship between dietary intake and neuropsychological outcomes (23, 24). It is also possible that, in people with diabetes, a higher HEI score may partly reflect disease-driven dietary modification after diagnosis, greater disease severity, or clinical counseling rather than a naturally protective lifestyle pattern. Unmeasured diabetes-related factors, including medication use, diabetes duration, glycemic control, complications, and post-diagnosis dietary changes, may also confound the association. Therefore, our findings highlight that the association between diet quality and depressive symptoms may differ according to metabolic status, but the diabetes interaction should be regarded as hypothesis-generating until confirmed in prospective and interventional studies.

This study has several strengths. The primary analysis was based on a large, nationally representative sample with rigorous adjustment for multiple confounders and appropriate consideration of the NHANES sampling design. HEI-2015, which is grounded in the Dietary Guidelines for Americans and can be derived from standardized dietary recall data, provided a comprehensive assessment of overall dietary quality rather than focusing on isolated nutrients or food items (25–27). We also examined depressive symptoms from both categorical and continuous perspectives by analyzing PHQ-9-defined clinically relevant depressive symptoms and PHQ-9 scores, respectively. In addition, subgroup analyses and spline models allowed us to explore effect modification and dose-response patterns in greater detail. The inclusion of an independent hospital-based replication cohort strengthened the assessment of reproducibility, particularly for the diabetes interaction, although it should not be interpreted as a fully equivalent replication because dietary assessment and population sampling differed between cohorts.

Several limitations should also be acknowledged. First, the cross-sectional design of both datasets precludes causal inference, and reverse causation cannot be excluded. Individuals with depressive symptoms or diabetes may change their diet after symptom onset or disease diagnosis, which could influence the observed associations. Second, PHQ-9 is a screening instrument rather than a clinical diagnostic interview for major depressive disorder. In addition, although PHQ-9 has been widely used internationally, cross-cultural measurement non-equivalence may exist when applying the same cut-off in U.S. and Chinese populations; differences in symptom expression, stigma, language, and response style may affect comparability between cohorts. Third, dietary intake in NHANES was assessed using 24-h recall and in the replication cohort through questionnaire-based dietary assessment, both of which may be subject to recall bias and measurement error. The hospital-based HEI-2015 was a harmonized approximation rather than an identical replication of the NHANES 24-h recall-based HEI-2015. Fourth, substantial exclusions due to missing PHQ-9, dietary, laboratory, and covariate information may have introduced selection bias. Because the available de-identified analytic files contained complete cases only, we could not conduct a formal included-versus-excluded comparison, multiple imputation, or inverse-probability weighting for missingness. Fifth, although we adjusted for a wide range of covariates, residual confounding by unmeasured factors remains possible, including antidepressant medication use, sleep quality, psychosocial stress, social support, dietary changes after diagnosis, diabetes duration, glycemic control, diabetes medications, and diabetes complications. Sixth, multiple subgroup analyses were performed, so subgroup findings other than the reproducible diabetes interaction should be interpreted as exploratory. Finally, although the spline analyses supported an overall association, they did not provide strong evidence for statistically significant nonlinearity, suggesting that the dose-response pattern should be interpreted cautiously.

From a clinical and public health perspective, our findings are consistent with previous evidence linking higher diet quality or dietary-guideline adherence to more favorable mental health-related outcomes and broader health benefits, suggesting that diet quality is relevant to depressive symptom burden, but they should not be interpreted as proving that improving HEI will prevent depression (28–31). The replicated interaction with diabetes suggests that associations between diet quality and mental health may need to be considered within a broader metabolic context. Future prospective studies are needed to clarify the temporal relationship between HEI and depressive symptoms, while mechanistic and interventional studies should further investigate why the association differs by diabetes status, including pathways related to high-fat or Western-style dietary exposure, oxidative stress, microbiota-gut-brain signaling, omega-3 fatty acids, fish intake, and B-vitamin status (32–42). Such work may help refine metabolically informed dietary strategies for improving mental health outcomes.

## 5 Conclusion

In conclusion, higher dietary quality, as measured by HEI-2015, was cross-sectionally associated with lower odds of PHQ-9-defined clinically relevant depressive symptoms and lower depressive symptom burden in the general adult population. These findings were observed in the nationally representative NHANES cohort and were further reproduced in an independent hospital-based replication cohort, supporting the consistency of the association across two datasets. Notably, diabetes consistently modified this relationship: higher HEI was associated with more favorable depression-related outcomes among individuals without diabetes, whereas the association was directionally opposite among those with diabetes. Given the cross-sectional design, potential reverse causation, measurement differences between cohorts, and residual confounding, these findings should be interpreted as hypothesis-generating and require confirmation in prospective and interventional studies.

## Data Availability

The original contributions presented in the study are included in the article/supplementary material, further inquiries can be directed to the corresponding author.
